# Aberrant NK cell profile in gestational diabetes mellitus with fetal growth restriction

**DOI:** 10.3389/fimmu.2024.1346231

**Published:** 2024-02-05

**Authors:** Yujing Xiong, Yazhen Wang, Mengqi Wu, Shuqiang Chen, Hui Lei, Hui Mu, Haikun Yu, Yongli Hou, Kang Tang, Xutao Chen, Jie Dong, Xiaohong Wang, Lihua Chen

**Affiliations:** ^1^Department of Immunology, Air Force Medical University, Xi’an, Shaanxi, China; ^2^Reproductive Medical Center, Department of Obstetrics and Gynecology, Tangdu Hospital, Air Force Medical University, Xi’an, Shaanxi, China; ^3^School of Life Science and Technology, ShanghaiTech University, Shanghai, China

**Keywords:** gestational diabetes mellitus, natural killer cell, fetal growth restriction, cell type, intrauterine infusion

## Abstract

Gestational diabetes mellitus (GDM) is a gestational disorder characterized by hyperglycemia, that can lead to dysfunction of diverse cells in the body, especially the immune cells. It has been reported that immune cells, specifically natural killer (NK) cells, play a crucial role in normal pregnancy. However, it remains unknown how hyperglycemia affects NK cell dysfunction thus participates in the development of GDM. In this experiment, GDM mice were induced by an intraperitoneal injection of streptozotocin (STZ) after pregnancy and it has been found that the intrauterine growth restriction occurred in mice with STZ-induced GDM, accompanied by the changed proportion and function of NK cells. The percentage of cytotoxic CD27^-^CD11b^+^ NK cells was significantly increased, while the proportion of nourished CD27^-^CD11b^-^ NK cells was significantly reduced in the decidua of GDM mice. Likewise, the same trend appeared in the peripheral blood NK cell subsets of GDM patients. What’s more, after intrauterine reinfusion of NK cells to GDM mice, the fetal growth restriction was alleviated and the proportion of NK cells was restored. Our findings provide a theoretical and experimental basis for further exploring the pathogenesis of GDM.

## 1 Introduction

Gestational diabetes mellitus (GDM) is a prevalent pregnancy complication characterized by elevated blood glucose levels that are first detected or recognized during pregnancy, and most of the abnormal glucose metabolism will recover after childbirth (1, 2). According to the International Diabetes Federation, GDM affects approximately 14% of pregnant women globally (3), with a prevalence rate in China of about 11.9%, which is increasing year by year (4, 5). GDM may lead to a series of adverse pregnancy outcomes including preterm birth, stillbirth, fetal growth restriction, low birth weight, or macrosomia (6–9). GDM is associated with some long-term diseases such as type-2 diabetes in both mother and child (6, 10). Therefore, GDM seriously affects maternal and fetal health, and it is meaningful to explore the mechanisms by which adverse pregnancy outcomes occur.

Numerous studies have been conducted to investigate the mechanisms underlying the development of gestational diabetes, with a consistent finding indicating that pregnant women who develop this condition exhibit impaired responsiveness of pancreatic beta cells to increased insulin demands during pregnancy, coupled with reduced insulin sensitivity, resulting in varying degrees of hyperglycemia (10, 11). Nevertheless, the mechanism of how elevated maternal glucose affects pregnancy outcomes remains unclear. There have been several studies recently that examine the relationship between immune dysfunction and GDM, suggesting that abnormalities in the number and function of immune cells may be associated with adverse pregnancy outcomes (12–14). For example, Lobo et al. found a significant decrease in regulatory T-cells (Treg) and a significant increase in cytotoxic natural killer (NK) cells (CD56^dim^CD16^+^) in the peripheral blood of patients with GDM (15).

During pregnancy, the placentation is crucial for ensuring fetal nutrition acquisition and maternal-fetal communication. Despite the different placental structures of humans and mice, the functions of placental villous units are similar (16). Previous studies showed that NK cells in placenta/decidua play a vital role in the development of the maternal-fetal immune microenvironment, in the maintenance of maternal-fetal immune tolerance, against infection and the spread of placental pathogens, as well as in the promotion of fetal growth and development (17, 18). Notably, NK cells in decidua present different phenotypes and functions from circulating NK cells, decidual NK cells could interact with the fetus by human leukocyte antigen (HLA) ligands expressed on extravillous trophoblasts (EVTs) to mediate immune tolerance between mothers and fetuses. Besides, decidual NK cells could secrete a variety of cytokines such as vascular endothelial growth factor, interleukin-8 (IL-8), and growth-promoting factors, associated with the ability to promote vascular remodeling, trophoblast invasion, and fetal development (19).

NK cells belong to innate lymphoid cells (ILCs) along with ILC1s, ILC2s, ILC3s, and lymphoid tissue-inducer (LTi) cells. NK cells are the most abundant leukocytes during pregnancy, while non-NK ILCs also exist in the uterus during the reproductive process with a low number (20). ILC1 is a subset that could produce interferon-γ (IFN-γ) and Th1-like cytokines; ILC2 is known to produce Th2-like cytokines and express of Gata3; ILC3 could produce IL-22 and IL-17; and LTis is important in secondary lymphoid organ formation (21). NK cells could be classified into different subsets according to their functions and the expression of markers on their surfaces, of which the CD56^dim^CD16^+^ and CD27^-^CD11b^+^ NK cells are mostly cytotoxicity, while CD56^bright^CD16^-^ and CD27^-^CD11b^-^ NK cells are mostly secretory and nourishing (22, 23). In addition, CD27^+^CD11b^-^ and CD27^+^CD11b^+^ NK cells are considered regulatory NK cells capable of secreting cytokines (24). The conversion of NK cell phenotype and function during pregnancy may result in adverse pregnancy outcomes, such as miscarriage or fetal growth restriction (25, 26). An understanding of how NK cells are altered in a high-glucose environment and how this may affect pregnancy outcomes remains to be determined.

Therefore, our study detected the phenotype and functions of maternal NK cells in mid- to late-term pregnancy in a high-glucose environment during pregnancy, and attempted to improve pregnancy outcomes through intrauterine immune perfusion of NK cells. We hope to find potential therapeutic approaches to rescue adverse pregnancy outcomes in GDM.

## 2 Materials and methods

### 2.1 Animal experiences

Seven to eight weeks male and female ICR mice were purchased from Tengxin Biotechnology Co., Ltd., Chongqing, China. Seventy female mice and sixteen male mice were used while breeding, and thirteen female mice were excluded from the study as they failed to pregnant. Mice were housed in a stable facility at 25°C with 12 h light/12 h dark cycles. All mice were stabilized for one week before experimental procedures. Female mice were mated with male mice over­night at a proportion of 1:1 per cage. Pregnancy was determined by the presence of vaginal plugs the next morning, which was identified as gestational day 0.5. Mice were randomly divided into the GDM and natural control (NC) groups. GDM mice were rendered hyperglycemic by an intraperitoneal injection of streptozotocin (STZ) (Sigma Chemical, St Louis, MO, USA), (50 mg/kg, dissolved in 0.1 mmol/L citrate buffer, pH 4.2–4.5), followed by 5 injections every 24 h (27). Previous studies indicated that STZ selectively destroys pancreatic β cells in animals, without a direct toxic effect on fetal growth (28–31). The same volume of citrate buffer was injected into NC mice. Random blood glucose was tested from the tail with a glucometer (Sinocare, Hunan, China) 72 h after the first STZ injection. Mice with random blood glucose ≥ 200 mg/dl were considered qualified GDM mice (4). Weight and blood glucose are monitored every two days after injection. The female mice and their fetuses were sacrificed at 14.5 days of gestation to evaluate the number of embryos implanted, the number of miscarriages, and the weight of the fetus and placenta. Immune cells were isolated from peripheral blood, placenta, and spleens. All experimental procedures involving animals were conducted in accordance with the Guide for the Care and Use of Laboratory Animals (China), with approval from the Air Force Medical University Experimental Animal Ethics Committee.

### 2.2 Patient recruitment and sample collection

We recruited ten GDM patients and ten healthy pregnant women with normal glucose tolerance at around 28 weeks gestation. The diagnosis of GDM was based on the International Panel on Diabetes and Pregnancy criteria, i.e., fasting blood glucose ≥92 mg/dl, 1-hour glucose level ≥180 mg/dl, or 2-hour glucose level ≥153 mg/dl in oral glucose tolerance test (OGTT). Patients under 18 years old, with preconception body mass index (BMI) >24 kg/m^2^, other types of diabetes, or with other pregnancy complications such as hypertension were excluded from the study.

We collected fasting peripheral blood from these pregnant women at the time of OGTT examination, and 5 ml of venous blood was drawn in tubes containing ethylene diamine tetraacetic acid (EDTA) anticoagulant. After that, peripheral blood mononuclear cell (PBMC) was isolated using a lymphocyte isolation solution (Dakewe Biotech Co., Ltd. Shenzhen, China), and the lymphocytes were collected, washed, and resuspended in PBS in preparation for flow cytometric staining.

### 2.3 Preparation of spleen cells and peripheral blood

Firstly, mouse spleen tissue was isolated and passed through a 70 mm mesh filter with a 5 ml syringe plunger, followed by centrifugation and washing to prepare single cell suspensions. Using red blood cell lysis (Yeasen Biotechnology Co., Ltd, Shanghai, China) to remove red blood cells from peripheral blood and spleen single-cell suspensions. The lysis was terminated with serum and washed twice in phosphate buffer solution (PBS) before being resuspended in a Flow Cytometry Staining Buffer for flow cytometry.

### 2.4 Isolation of placental immune cells

We carefully dissected pregnant mouse placentas and separated them from the fetus. Afterward, the placentas were washed twice in ice-cold PBS. The placentas of each GDM dam were collected, cut into 1 mm^3^ pieces, and enzymatically digested in 1640 medium containing 0.1% type IV collagenase (Sigma-Aldrich Corp, St. Louis, MO, USA) and 0.01% DNase I (Sigma-Aldrich Corp.) at 37°C for one hour. Following filtering through cell strainers and centrifugation, the pellets were resuspended in RPMI 1640 medium. Density gradient centrifugation was used to isolate individual immune cells. The solutions of Percoll (Yeasen Biotechnology Co.) were prepared at three different densities: 20%, 40%, and 60%. The cell suspension was lightly added to the Percoll solution and centrifuged. Between the 40% and 60% Percoll layers, cells were collected, washed twice, and then resuspended in RPMI 1640 medium.

### 2.5 Isolation of NK cells

We chose pregnant mice as donors and obtained immune cells from their spleens. The NK cells were isolated using the MojoSort Mouse NK Cell Isolation Kit (Biolegend, San Diego, CA, USA) according to the manufacturer’s instructions. In brief, the filtered splenocytes were resuspended in a volume of buffer to give a final concentration of 10^8/ml. Transfer 100 μl splenocyte suspension into a new sterile tube, add 10 μl NK Cell Biotin-Antibody Cocktail, and then mix well samples and store on ice for 15 minutes. After washing and centrifugation, discard the supernatant and add 10 µL Anti-Biotin MicroBeads, mix well and incubate for additional 15 minutes on ice, and then wash twice. Finally, the beads and cells are reselected and placed in a magnetic separator for 5 minutes, the target cell solution is poured out, and the solution obtained is the purified NK cell suspension by repeating the process 2-3 times.

### 2.6 Injections of NK cells in the uterine horn

Purified NK cells (resuspended in PBS) were supposed to be injected into the uterine horn in the treatment group, and the same volume of PBS was supposed to be injected into the uterine horn in the control group. The operation is performed at 2.5 days of gestation. The mice were anesthetized by isoflurane, and the fat pads on the ovaries were exposed by undercutting the mice in the dorsal midline. We gently removed the fat pad from the uterus and secured it with vascular clamps. In the following steps, NK cells and PBS were injected into the proximal endometrium near the oviduct using an oral pipette, and the skin incision was closed with 6-0 nylon sutures. 5×10^5 cells were injected per side/mouse. After completing the procedure, the mice were placed on a heating pad until they were fully awake.

### 2.7 Flow cytometry

Cell surface staining was conducted with the appropriate fluorochrome-conjugated antibodies for 30 min at 4°C in dark. After fixation and permeabilization with Intracellular (IC) Fixation Buffer (Invitrogen, Carlsbad, CA, USA) and Permeabilization Buffer (Invitrogen) according to the manufacturer’s protocol, intracellular staining was conducted with the appropriate fluorochrome-conjugated Abs for 30 min at 4°C. The following specific monoclonal antibodies were used: PerCP-conjugated anti-mouse CD45 (#103130, clone 30-F11), APC/Cyanine7-conjugated anti-mouse CD45 (#103154, clone 30-F11), PE/Cyanine7- conjugated anti-mouse CD3 (#100220, clone 17A2), FITC-conjugated anti-mouse NK1.1 (#108706, clone PK136), PerCP- conjugated anti-mouse NK1.1 (#108727, clone PK136), APC/Cyanine7-conjugated anti-mouse/human CD27 (#124226, clone LG.3A10), PE-conjugated anti-mouse/human CD11b (#101208, clone M1/70), PE-conjugated anti-mouse Perforin (#154305, clone S16009A), FITC-conjugated anti-mouse Granzyme B (#515403, clone GB11), APC-conjugated anti-mouse IFN-γ (#505810, clone XMG1.2). FITC-conjugated anti-human CD45 (#304006, clone HI30), PE/Cyanine7- conjugated anti-human CD3 (#300420, clone UCHT1), PE-conjugated anti-human CD56 (#304606, clone MEM-188), Pacific Blue-conjugated anti-human CD16 (#302032, clone 3G8), APC-conjugated anti-human CD226 (#338312, clone 11A8), APC/Fire™ 750-conjugated anti-human NKG2D (#320834, clone 1D11), and PerCP, PE, PE/Cyanine7, FITC, APC, APC/Cyanine7, Pacific Blue conjugated Isotype (all purchased from Biolegend, San Diego, CA, USA). We used PBS containing 0.1% NaN3 and 2% FBS as FACS buffer. Flow cytometric analysis was performed using a flow cytometer (ACEA, Carpinteria, CA, USA). We stained a single sample of cells with antibodies that bind to exclusive populations for generating compensation controls.

### 2.8 H&E staining

The placenta was peeled from the uterus, immediately fixed in 4% paraformaldehyde (Beyotime Biotechnology, Shanghai, China), and embedded in paraffin, the maximum cross-section was selected and cut into 5-μm-thick sections. The sections were deparaffinized with de-paraffin liquid (Servicebio, Wuhan, China) and dehydrated using gradient alcohol. Subsequently, the sections were stained with hematoxylin (Servicebio) for 5 min and followed by eosin staining (Servicebio) for 3 min. After staining, the slices were transparent with anhydrous ethanol, n-butanol, and xylene, and sealed with neutral gum. The images of stained sections were visualized with a microscope (Nikon, Tokyo, Japan).

### 2.9 Public data collection and pseudo-time analysis

Raw data for pseudo-time analysis were obtained from publicly available datasets: Gene Expression Omnibus database and the Genomic Spatial Event (GSE) 173193. Pseudotime analysis was performed on NK cells using Monocle Monocle 2.24.0. We used the differential GeneTest function (fullModelFormulaStr = “∼clusters”) of the Monocle 2.24.0 package for ordering genes (qval < 0.01) that may be informative for sorting cells along pseudo time trajectories. The ordered genes were then marked with the setOrderingFilter function. Gene expression was then plotted as a function of pseudo-time in Monocle 2.24.0 to track changes across pseudo-time.

### 2.10 Statistical analysis

Prism 8.4.3 software (GraphPad) was used for data analysis. The Shapiro-Wilk test was used to determine the normality of continuous variables. Statistical significance was determined using Student’ s t-test or Mann-Whitney U test for two groups. Correlation analysis involved the utilization of Spearman correlation. One-way ANOVA and Tukey’s *post hoc* test was used for multiple group comparisons. The data are presented as mean ± SEM. Statistical significance was defined as *P*<0.05.

## 3 Result

### 3.1 Hyperglycemia affects pregnancy outcomes in the non-obese GDM mouse model

**Figure 1:**
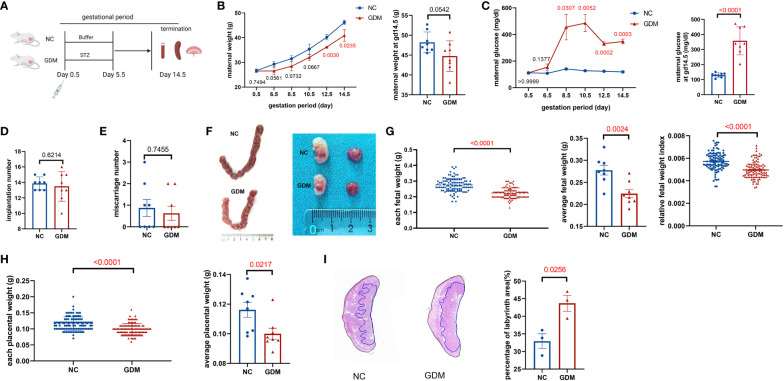
Experimental protocol for GDM mouse models and the pregnancy outcomes of normal and GDM mouse models. **(A)** Schematic diagram showing the experimental design of the mouse model establishment. Mice were killed on day 14.5 after mating, pregnancy outcomes were observed and immune cells were isolated and analyzed. **(B)** Maternal weight and **(C)** blood glucose in normal pregnancy or GDM pregnancy were measured during gestation (dams: n=3 each group) and at GD14.5 (dams: n=8 each group). All dams were killed on GD14.5, **(D)** the implantation numbers and **(E)** miscarriage numbers were presented. **(F)** Representative picture of uterus and fetuses from normal pregnancy and GDM pregnancy at GD14.5. **(G)** Each fetal weight, average fetal weight of each GDM dam and relative fetal weight index (fetal weight/maternal weight) was significantly decreased than normal dam, as well as **(H)** each placental weight and average placental weight of each GDM dam. Fetus: n=103 in normal pregnant group, n=104 in GDM pregnant group, dams: n=8 each group. **(I)** Representative picture of placental HE staining in NC and GDM dams, the proportion of labyrinth area of GMD was significantly higher than that in NC group, dams: n=3 each group. Data are presented as mean ± SEM. Mann-Whitney U tests were used for comparison of miscarriage number, each fetal weight, and each placental weight, and Student’s t-tests were used to calculate other values. (Figure A was created with Biorender.com).

To observe the alteration of pregnancy outcome in the GDM mouse model, we constructed a post-pregnancy hyperglycemic mouse model by intraperitoneal injection of STZ specifically damaging pancreatic islet cells after mating (**Figure 1A**). The body weight of pregnant mice gradually increased with gestation advancement, but the increase in body weight of non-obese GDM mice was smaller than that of normal pregnancy (**Figure 1B**), and maternal blood glucose increased significantly from gestation day 8.5 onwards in GDM mice (**Figure 1C**). The mice were sacrificed on day 14.5 of gestation, and the weight of GDM model pregnant mice (44.75 ± 1.37 g vs. 48.22 ± 0.92 g, *P*=0.0542) was lower than that of normal pregnant mice (**Figure 1B**), and the blood glucose (358.0 ± 32.93 mg/dl vs. 130.3 ± 5.24 mg/dl, *P*<0.0001) was significantly higher than that of normal pregnancy (**Figure 1C**), which indicates the non-obese GDM mouse model was constructed successfully.

At 14.5 days of gestation, further observation of the pregnancy outcome of the GDM mouse model revealed a similar frequency of implantations (13.50 ± 0.68 vs. 13.88 ± 0.30, *P*=0.6214) and miscarriages (0.63 ± 0.32 vs. 0.87 ± 0.40, *P*=0.7455) in both groups of mice (**Figures 1D, E**). Moreover, the fetus in the non-obese GDM model was smaller than in normal pregnancies (**Figure 1F**), we also found that the average baby weight (0.22 ± 0.01 g vs. 0.27 ± 0.01 g, *P*=0.0024) and placenta weight (0.10 ± 0.004 g vs. 0.12 ± 0.005 g, *P*=0.0217) were lower than normal (**Figures 1G, H**). HE staining of the placenta revealed a significant increase in the labyrinthine layer in GDM mice (43.69 ± 2.26% vs. 32.92 ± 2.14%, *P*=0.0256), suggesting abnormal placental function (**Figure 1I**). According to the results of this study, the environment of high glucose in pregnancy may have contributed to fetal growth restriction, in agreement with previous findings (32).

### 3.2 Hyperglycemia alters immune homeostasis, including NK cell proportions and functions

**Figure 2:**
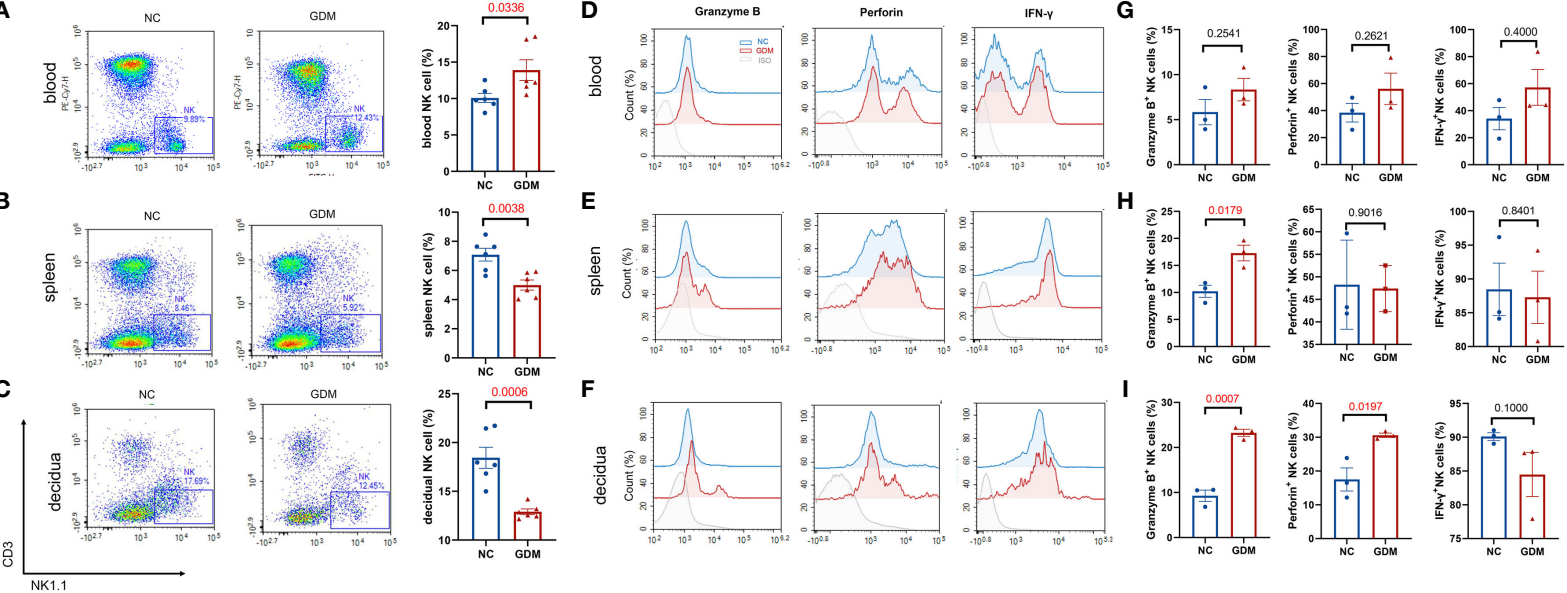
High glucose environment altered NK cell proportion and function in GDM mice. Termination of pregnancy on day 14.5, cells from deciduas, spleens and blood were collected and analyzed by flow cytometry. Representative and quantitative results for NK cells in the **(A)** blood, **(B)** spleen, and **(C)** decidua derived from normal dams and GDM dams. Dams: n=6 each group. Function related molecules (Granzyme B, Perforin, IFN-γ) were analyzed, representative images showing NK functional molecular changes in peripheral blood, spleen, and decidua between GDM mice and normal pregnant mice **(D–F)**, statistical charts showing the results in the **(G–I)**. Dams: n=3 each group. Data are presented as mean ± SEM. Mann-Whitney U tests were used for comparison of IFN-γ in blood and decidua, and Student’s t-tests were used to calculate other values.

It has been reported that continuous exposure to hyperglycemia induces a low-grade inflammatory state in the body (33), and in pathological conditions such as GDM, the immune homeostatic balance at the maternal-fetal interface is disrupted and placental function is impaired (34). Since NK cells are widely studied and considered to be one of the most critical immune cells during pregnancy, we wanted to observe the changes in peripheral blood, splenic, and decidual NK cells under a high glucose environment. The immune cells of pregnant mice were collected at gestational day (GD)14.5 and analyzed. **Supplementary Figure 1** illustrates the circle-gate strategy of flow cytometry, where peripheral blood NK cells were labeled as CD3^-^NK1.1^+^, while splenic and decidual NK cells were labeled as CD45^+^CD3^-^NK1.1^+^. According to flow cytometry results (**Figures 2A–C**), the average percentage of NK cells in peripheral blood was significantly higher in GDM mice (13.90 ± 1.42% vs. 10.07 ± 0.63%, *P*=0.0336) than in normal pregnant mice. In contrast, the splenic NK cell percentage (5.00 ± 0.34% vs. 7.08 ± 0.44%, *P*=0.0038) and decidual NK cell percentage (12.90 ± 0.30% vs. 18.44 ± 1.08%, *P*=0.0006) were significantly lower than normal pregnant mice. It was reported that adverse pregnancy was associated with enhanced cytolysis in NK cells (35), the cytotoxic ability and the secretion of pro-inflammatory cytokine IFN-γ were subsequently investigated by flow cytometry. Peripheral blood granzyme B^+^ (8.34 ± 1.24% vs. 5.84 ± 1.41%, *P*=0.2541), perforin^+^(56.09 ± 11.64% vs. 38.44 ± 6.91%, *P*=0.2621), and IFN-γ^+^ NK cells (57.17 ± 13.23% vs. 34.11 ± 8.23%, *P*=0.4000) showed an increasing trend in GDM mice, but there were no statistical significances (**Figures 2D, G**); splenic NK cells in GDM mice expressed higher granzyme B (17.29 ± 1.45% vs. 10.21 ± 1.11%, *P*=0.0179) (**Figures 2E, H**). It is worth noting that cytotoxic NK cells in decidua (granzyme B: 23.25 ± 0.78% vs. 9.29 ± 1.25%, *P*=0.0007, perforin: 30.58 ± 0.64% vs. 17.50 ± 3.42%, *P*=0.0197) significantly increased in hyperglycemia (**Figures 2F, I**), indicating that those expressing immune tolerant NK cells at maternal fetal interface inverted into higher cytotoxicity under a high glucose environment, which might compromise pregnancy.

### 3.3 The proportion and spatial distribution of NK cell subsets are changed in the non-obese GDM mouse model

**Figure 3:**
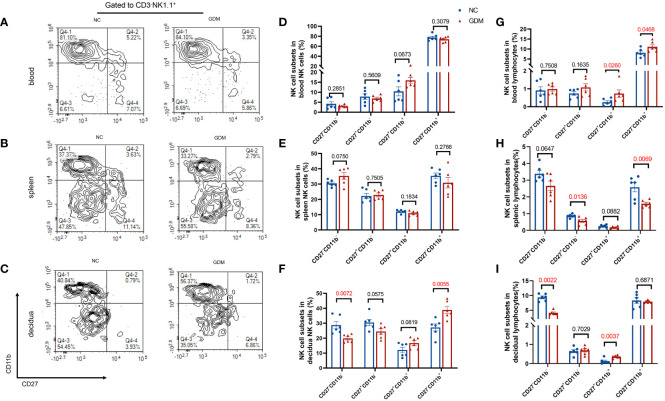
The proportion of CD27^-^CD11b^-^ NK cells decreased and the proportion of CD27^-^CD11b^+^ NK cells increased in decidua in GDM mice. NK cells were categorized into four cell subsets based on the expression of CD27 and CD11b. Representative flow cytometry result for CD27 and CD11b expression in **(A)** blood, **(B)** spleen, **(C)** decidual NK cells. **(D–F)** Quantitative results for NK cell subsets in total NK cells in blood, spleen, and decidua. **(G–I)** Proportions of different NK cell subsets in total lymphocytes in blood, spleen, and decidua. Data are presented as mean ± SEM. Dams: n=6 each group. Mann-Whitney U tests were used for comparison of CD27^+^CD11b^+^ cells proportion in blood lymphocyte, CD27^-^CD11b^-^ cells proportion in spleen NK cells, and CD27^-^CD11b^-^ in decidual lymphocytes, and Student’s t-tests were used to calculate other values.

NK cells could be classified into four subpopulations based on their expression density of CD27 and CD11b surface antibodies (36), a scheme of the NK cell subsets was shown in **Supplementary Figure 1D**. Thus, we investigated the distribution of NK cell subpopulations in peripheral blood, spleen, and decidua of GDM mice. As shown in **Figures 3A–C**, representative figures of NK cell subpopulations from peripheral blood, spleen, and decidua of the two groups were presented. Approximately 80% of the peripheral blood NK cells were CD27^-^CD11b^+^ type, while the majority of NK cells in the spleen and decidua were CD27^-^CD11b^-^ type.

The composition of peripheral blood and spleen NK cells did not show significant differences between GDM mice and normal pregnant mice (**Figures 3D, E**). However, there was a notable decrease in the percentage of CD11b^-^CD27^-^ (19.88 ± 1.10% vs. 28.75 ± 2.40%, *P*=0.0072) in the decidua, while an increase was observed in the CD11b^+^CD27^-^ subtype (38.91 ± 2.35% vs. 27.19 ± 2.36%, *P*=0.0055) within the decidua (**Figure 3F**). In terms of total lymphocytes, CD27^-^CD11b^+^ (11.11 ± 1.03% vs. 8.11 ± 0.83%, *P*=0.0468) and CD27^+^CD11b^+^ subsets (0.73 ± 0.21% vs. 0.25 ± 0.08%, *P*=0.0260) were significantly higher in GDM mice peripheral blood (**Figure 3G**), CD27^-^CD11b^+^ (1.59 ± 0.09% vs. 2.58 ± 0.28%, *P*=0.0069) and CD27^+^CD11b^-^ (0.57 ± 0.09% vs. 0.86 ± 0.04%, *P*=0.0136) subsets were decreased in the spleen (**Figure 3H**), and at the maternal-fetus interface, CD11b^-^CD27^-^ NK cells were significantly decreased (4.13 ± 0.29% vs. 9.36 ± 0.48%, *P*=0.0022) (two-fold), whereas CD27^+^CD11b^+^ subsets increased (0.36 ± 0.03% vs. 0.12 ± 0.05%, *P*=0.0037) (**Figure 3I**).

### 3.4 A correlation exists between the proportion of NK cells in the decidua and fetal weight

**Figure 4:**
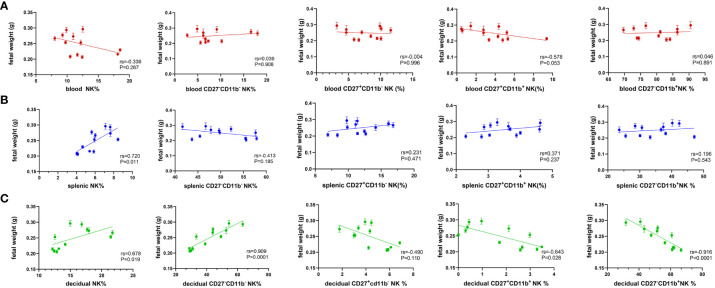
Decidual NK subsets show a strong correlation with fetal weight. Spearman correlation analysis was used to describe the relationship between the proportion of NK cells in total lymphocyte, the proportion of CD27^-^CD11b^-^, CD27^-^CD11b^+^, CD27^+^CD11b^+^, CD27^+^CD11b^-^ in total NK cells in **(A)** blood, **(B)** spleen and **(C)** decidua with fetal weight. Dams: n=12.

As shown above, maternal hyperglycemia during pregnancy influenced the proportion and composition of circulating and local NK cells. The relationship between NK cell content in different parts of the mother and fetal weight was further examined. **Figure 4A** indicates that the proportion of NK cells in peripheral blood was negatively correlated with fetal weight, whereas the total number of NK cells in the spleen was positively correlated with fetal weight (**Figure 4B**). Total NK cells in the decidua are positively correlated with fetal weight, whereas CD27 single-positive NK cells and double-positive NK cells are negatively linked. More importantly, in **Figure 4C**, CD27^-^CD11b^–^type NK cells were strongly positively correlated with fetal weight, while CD27^-^CD11b^+^- type NK cells were strongly negatively correlated (RS>0.9). Hence, this result suggests a stronger correlation between decidual NK cell subpopulations and fetal growth potential.

### 3.5 Intrauterine NK cell immunotherapy improves pregnancy outcome in GDM pregnant mice

Recently, intrauterine immune cells/cytokine infusions have received increasing attention in the field of reproduction, and have been used to treat a variety of adverse pregnancies (37). Based on our findings that abnormal NK cell proportions at the maternal-fetal interface related to adverse pregnancy outcomes in GDM mice, we conducted an experimental design to determine whether intrauterine NK cell immunotherapy could improve pregnancy outcomes for GDM babies. We performed the immunotherapy on the 2.5th day following mating and isolated NK cells from the spleens of normal pregnant mice.

**Figure 5:**
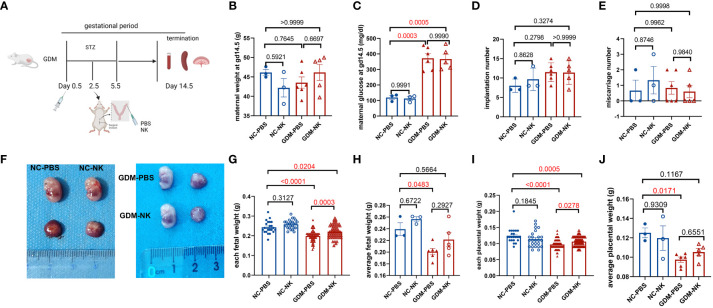
Pregnancy outcomes at 14.5 days of gestation after intrauterine NK cell infusion in NC and GDM mice. **(A)** Schematic representation of the construction of a mouse model for intrauterine immunotherapy, in which normal mouse-resident NK cells (from the spleen)/PBS were injected into the uterine horns of mice on day 2.5 after mating. Mice were killed on day 14.5 after mating, pregnancy outcomes were observed and immune cells were isolated and analyzed. **(B)** Maternal weights were similar among NC-PBS, NC-NK, GDM-PBS, and GDM-NK groups. **(C)** Blood glucose on GD14.5 was similar in the GDM-PBS and GDM-NK groups, while were significantly higher than NC groups. **(D)** There were no significant differences in implantation numbers and **(E)** miscarriage numbers among the four groups. **(F)** Representative picture of fetuses from NC-PBS, NC-NK, GDM-PBS and GDM-NK pregnancy at GD14.5. **(G)** Each fetal weight and **(I)** each placental weight in the therapy group increased in GDM, while still lower than NC groups, **(H)** average fetal weight and **(J)** average placental weight of each therapy dam also increased in GDM, but not statistically significant. Dams: n=3 in NC-PBS and NC-NK group, n=6 in GDM-PBS group, and n=5 in GDM-NK group. Fetus: n=22 in NC-PBS group, n=25 in NC-NK group, n=64 in GDM-PBS group, and n=54 in GDM-NK group. Data are presented as mean ± SEM. One-way ANOVA and Tukey’s *post hoc* test was used for multiple comparisons.(Figure A was created with Biorender.com).

Purified NK cells were injected into recipients by uterine horn injection, and the recipients were sacrificed on day 14.5 of gestation to extract and analyze immune cells from their peripheral blood, spleens, and decidua (**Figure 5A**). It was found that maternal weight, number of implantations, and number of abortions after intrauterine NK cell immunotherapy in GDM or normal pregnant mice were similar to those in the untreated groups (**Figures 5B, D, E**). Maternal blood glucose levels were similar in GDM-NK group and GDM-PBS group and were higher than normal pregnancy groups (**Figure 5C**). After intrauterine NK cell infusion, both fetal weight (0.22 ± 0.004 g vs. 0.20 ± 0.003 g, *P*=0.0003) and placental weight (0.11 ± 0.002 g vs. 0.10 ± 0.002, *P*=0.0278) were significantly elevated compared to the untreated group in GDM dams (**Figures 5F–J**), while each fetal weight and each placental in GDM-NK group remained lower than that of the normal pregnant mice (0.22 ± 0.004 g vs. 0.24 ± 0.007 g, *P*=0.0204; 0.11 ± 0.002 g vs. 0.12 ± 0.005 g, *P*=0.0005, respectively), suggesting that intrauterine NK cell infusion improves pregnancy outcomes in GDM mice, but it could not restore to the normal level. The results also showed that intrauterine infusion did not affect pregnancy outcomes in normal pregnancies (**Figures 5B–J**).

### 3.6 Intrauterine infusion modulates systemic and local NK cell proportions and subsets in GDM mice

**Figure 6:**
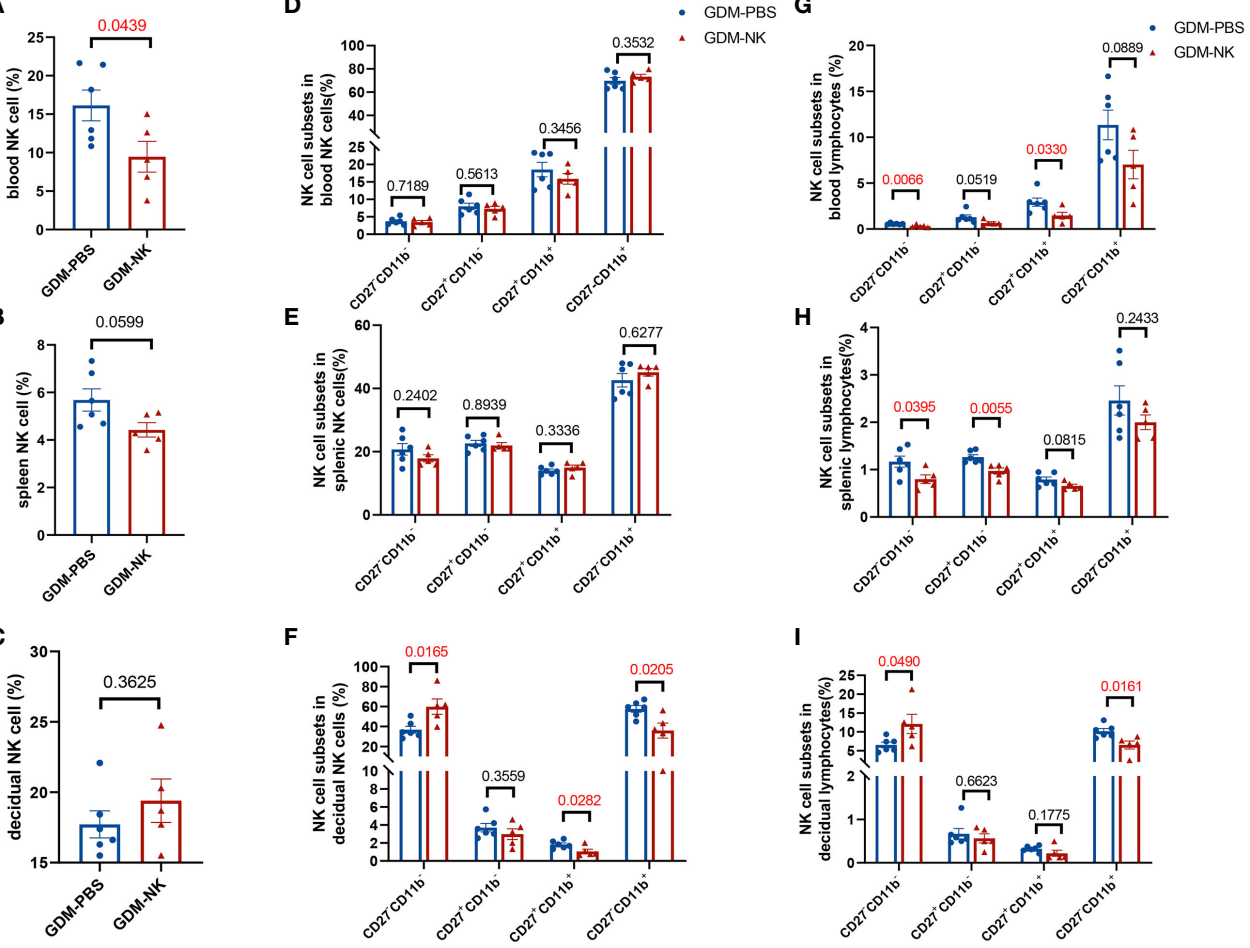
The proportion of CD27^-^CD11b^-^ NK cells increased and the proportion of CD27^-^CD11b^+^ NK cells decreased in decidua after intrauterine NK cell infusion in GDM mice. On GD14.5, cells from deciduas, spleens and blood were collected and analyzed by flow cytometry. **(A–C)** Total NK cell proportions in the blood, spleen, and decidua derived from therapy GDM dams and non-treated GDM dams. **(D–F)** Quantitative results for NK cell subsets in total NK cells in blood, spleen, and decidua. **(G–I)** Quantitative results for NK cell subsets in total lymphocytes in blood, spleen, and decidua. Dams: n=6 in GDM-PBS group, and n=5 in GDM-NK group. Data are presented as mean ± SEM. Mann-Whitney U tests were used for comparison of CD27^+^CD11b^-^ cells proportion, CD27^-^CD11b^+^ cells proportion in spleen NK cells, CD27^+^CD11b^-^ cells proportion in blood lymphocytes, CD27^+^CD11b^-^ cells proportion and CD27^+^CD11b^+^ in decidual lymphocytes, and Student’s t-tests were used to calculate other values.

The previous results indicated that the percentage and subpopulation compositions of NK cells were related to fetal growth potential, especially decidual NK cells. To determine whether immunotherapy improves pregnancy outcomes by modulating NK cells, we examined NK cells’ performance after treatment. The percentage of circulating NK cells was found to be significantly reduced following immunotherapy (9.47 ± 2.00% vs. 16.14 ± 2.00%, *P*=0.0439), reaching a level comparable to that observed in normal pregnant mice. However, no significant alterations were observed in the percentage of splenic (4.42 ± 0.30% vs. 5.68 ± 0.47%, *P*=0.0599) and decidual NK cells (19.40 ± 1.55% vs. 17.72 ± 0.96%, *P*=0.3625) (**Figures 6A–C**). As for the four subsets of NK cells, there were no significant changes in peripheral blood and spleen (**Figures 6D, E**), whereas the decidual NK cell subtypes experienced substantial changes, including a substantial increase in the proportion of CD27^-^CD11b^-^ cells (59.94 ± 7.64% vs. 36.80 ± 3.44%, *P*=0.0165) and a significant decrease in CD27^-^CD11b^+^ cells (36.00 ± 7.57% vs. 57.67 ± 3.26%, *P*=0.0205) and CD27^+^CD11b^+^ cells (1.06 ± 0.24% vs. 1.83 ± 0.18%, *P*=0.0282) (**Figure 6F**). After treatment, the proportion of double negative NK cells in lymphocytes decreased in blood and spleen but increased in decidua. The percentage of double-positive NK cells in blood, CD27 single-positive NK cells in spleen, and CD11b single-positive NK cells in decidua showed a significant decrease in lymphocytes. (**Figures 6G–I**).

Furthermore, intrauterine immunotherapy did not change the percentage of NK cells in the circulation, spleen, or decidua in normal pregnant mice (**Supplementary Figures 2A–C**), nor did NK cell subsets in spleen or decidua (**Supplementary Figures 2E–I**). However, blood CD27^+^CD11b^+^ NK cells were reduced while CD27^-^CD11b^-^ NK in lymphocytes were increased after treatment (**Supplementary Figures 2D, G**).

### 3.7 Increased cytotoxic NK cells in GDM patients

**Figure 7:**
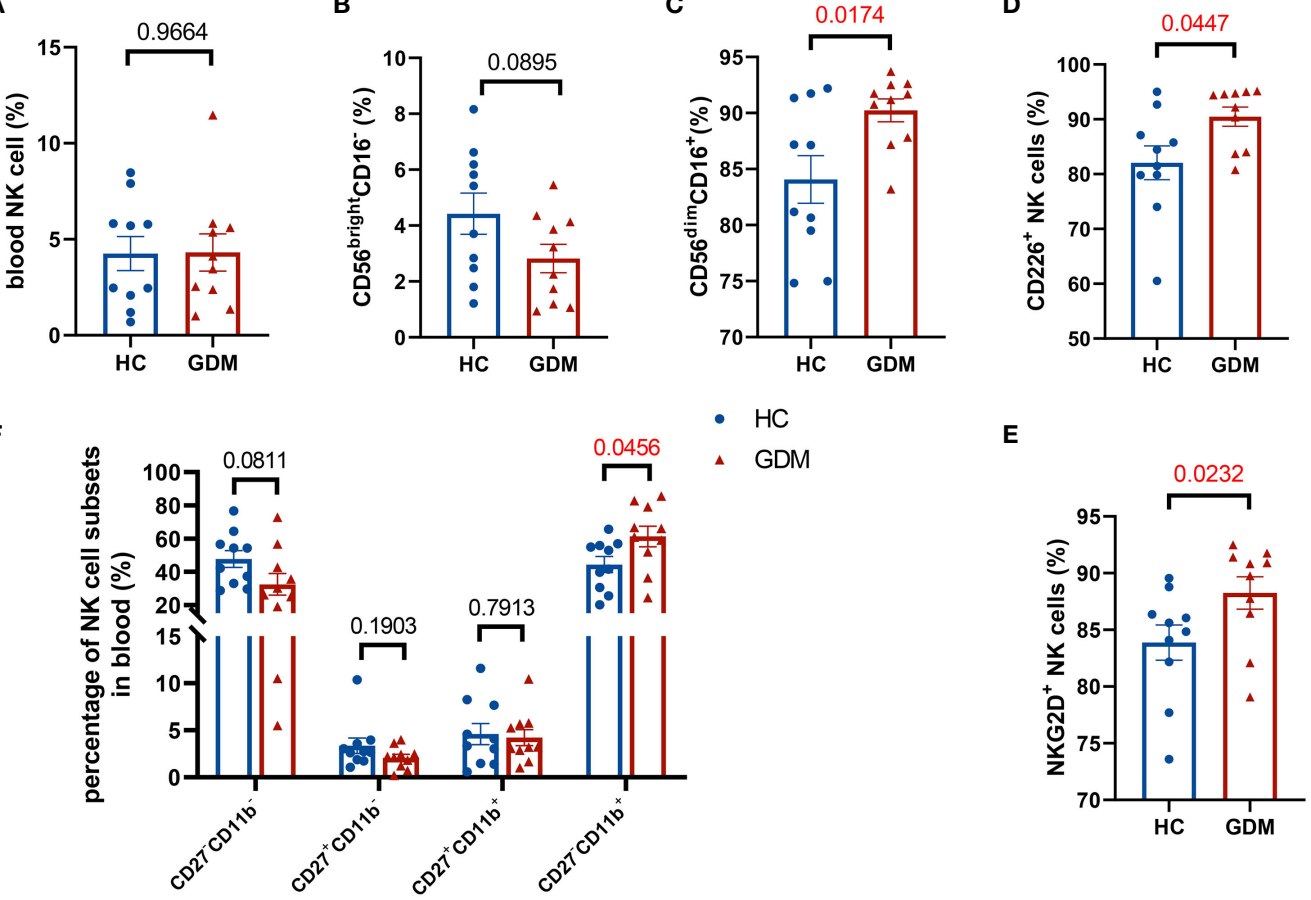
The percentage of cytotoxic NK cells in peripheral blood of GDM patients significantly increases. **(A)** There were no significant differences in the percentage of total NK cells between GDM patients and health control (HC) women. **(B)** The percentage of CD56^bright^CD16^-^ NK cells decreased and **(C)** CD56^dim^CD16^+^ NK cells significantly increased in GDM patients. **(D–F)** Percentage of CD226^+^ NK cells, NKG2D^+^ NK cells and CD27^-^CD11b^+^ NK cells significantly increased in GDM patients. Patients: n=10 for each group. Data are presented as mean ± SEM. Mann-Whitney U tests were used for comparison of CD226^+^, NKG2D^+^, CD27^+^CD11b^-^ NK cells proportions, and Student’s t-tests were used to calculate other values.

We recruited ten GDM patients without obesity and ten healthy pregnant women without GDM, and their clinical characteristics did not show any significant differences (**Table 1**). Peripheral blood was extracted from pregnant women and NK cells were detected in different phenotypes. Gating strategy of flow cytometry were shown in **Supplementary Figure 3**. There were no significant differences in the percentage of total NK cells between the two groups (4.31 ± 0.96% vs. 4.26 ± 0.89%, *P*=0.9664 in **Figure 7A**), while the proportion of CD56^bright^CD16^-^ NK cells was lower in GDM patients than in healthy pregnant women (2.82 ± 0.51% vs. 4.42 ± 0.74%, *P*=0.0895 in **Figure 7B**). Intriguing results revealed that peripheral blood cytotoxic NK cell subsets (CD56^dim^CD16^+^, CD226^+^, NKG2D^+^, CD27^-^CD11b^+^) were significantly higher in patients with GDM (90.24 ± 1.02% vs. 84.07 ± 2.12%, *P*=0.0174; 90.48 ± 1.76 vs. 82.07 ± 3.10, *P*=0.0447; 88.25 ± 1.42 vs. 83.88 ± 1.56, *P*=0.0232; 61.27 ± 6.24% vs. 44.35 ± 4.82%, *P*=0.0456 respectively in **Figures 7C–F**), suggesting that circulating NK cells in GDM patients were in an abnormally activated state.

**Table 1:** Demographic and clinical characteristics of GDM patients and HC.

Characteristics	HC (n=10)	GDM (n=10)	t	P
Maternal age (year)	33.4±3.24	32.9±3.21	0.347	0.777
Pre-gestational BMI (kg/m^2^)	22.09±2.07	21.08±2.60	0.960	0.502
Weight gain during gestation (kg)	15.45±4.02	15.3±2.63	0.099	0.152
Gestational age at delivery (week)	37.2±2.35	37.5±1.78	-0.322	0.523
Birth weight (g)	3150±254.95	3055±267.13	0.814	0.953

HC, health control; GDM, gestational diabetes mellitus.

## 4 Discussion

Our study constructed a non-obese GDM mouse model in order to examine the proportion, function, and subset composition of NK cells in circulating and at maternal-fetal interfaces under hyperglycemic conditions. We observed a significant increase in the percentage of NK cells, particularly CD11b^+^ NK cells in the peripheral blood of GDM mice. However, the proportion of NK cells in the spleen and decidua tissues of GDM mice was significantly reduced, with the most significant decrease in CD27^-^CD11b^+^ in the spleen, and a considerably decrease of CD27^-^CD11b^-^ NK cells in the decidua. Furthermore, CD27^-^CD11b^+^ NK cells are significantly increased in decidua, accompanied by enhanced cytotoxic function. Additionally, we observed that the proportion of different subsets of decidual NK cells was closely related to fetal weight, so we suspect intrauterine infusion of NK cells could enhance the immune microenvironment at maternal-fetal interfaces. This hypothesis has been partially supported by our study.

GDM may elevate the risk of various short-term and long-term maternal and fetal health complications (38). Recent studies indicated an increased likelihood of preeclampsia, surgical delivery, and subsequent development of type 2 diabetes mellitus in GDM moms (39). Besides, infants born to GDM mothers are at risk for abnormal intrauterine nutritional uptake (excessive/macrosomia or insufficient/low birth weight), stillbirth, and future onset of type 2 diabetes mellitus (40, 41). It has been reported that preeclampsia was associated with the shift to a cytotoxic subset in NK cells, which may lead to apoptosis of cytotrophoblasts and inhibit trophoblast invasion (42). Combining previous researches with our study, we suppose that an increase in the proportion of cytotoxic NK cells in GDM patients may be one of the reasons for the subsequent increase in the incidence of preeclampsia. Previous studies showed that GDM is caused by pancreatic dysfunction, where untimely insulin secretion in the mother’s body leads to glucose buildup, which triggers a range of adverse effects (11). There are some conflicting findings in the literature about fetal weight in GDM mice. Some studies reported that higher fetal weight or macrosomia were observed in GDM mice, studies using high-fat diets to construct obese GDM mice have found that altered glucose, amino acid, and fatty acid transport in the placenta stimulates the release of endogenous insulin-like growth factor-1 by the fetus, resulting in fetal overgrowth, which is an explanation to some extent for the development of macrosomia in overweight GDM patients (11, 43, 44). In contrast, other studies showed fetal weight was decreased in GDM mice (45), and a large clinical study of 11,486 pregnant women found that newborns with GDM had a 1.6 times higher risk of low birth weight than normal pregnant women (8), but the mechanism by which this occurs has not yet been identified. Our study has developed non-obese GDM mouse models whose fetuses exhibit a lower weight phenotype, which can be used as a research model to study fetal nutrition in relation to GDM. Moreover, a non-obese GDM mouse model may more intuitively reflect the impact of elevated blood glucose on pregnancy outcomes, ruling out the interference of metabolic diseases such as obesity.

There is a compelling evidence suggesting that hyperglycemia can lead to immune dysfunction (46), abnormal ratios of immune cells, altered secretion of cytokines, and an inflammatory state, thereby rendering the patient more susceptible to infection and related comorbidities (47, 48). Maternal hyperglycemia may trigger a “glucose stress” response and systemic inflammatory response, including changes in the infiltration, differentiation, and activation of maternal innate and adaptive immune cells (49). Moreover, cytokine expressions at the maternal-fetal interface also changed a lot under a high glucose environment, including an increase of IL-4, IL-6, IL-10, IL-17, and IFN-γ and a decrease of IL-1β and IL-8 (50). Imbalanced cytokine levels and inflammatory status may lead to NK dysfunctions. During pregnancy, the maternal immune system undergoes modifications to accommodate the fetus (51), and the imbalance between innate and adaptive cellular responses will pose an additional health threat to women with GDM (12, 52). It is believed that aberrant adaptation of maternal immune cells plays a critical role in low-grade inflammation and adverse maternal health outcomes linked to GDM diagnosis (12). Our study corroborates this notion by observing changes in proportions and functional subpopulations of NK cells at both circulating and maternal-fetal interfaces of GDM dams or patients.

NK cells could be divided into different subpopulations with distinct functions by different receptor expression (53). Human NK cells are mainly classified into CD56^dim^CD16^+^ and CD56^bright^CD16^-^ subsets based on the surface expression of CD56 and CD16, CD56^dim^CD16^+^ subset is more naturally cytotoxic while CD56^bright^CD16^-^ subset has the capacity to produce abundant cytokines (54, 55). According to their differentiation and developmental pathways, mice NK cells can generally be divided into four subpopulations, including CD27^-^CD11b^-^, CD27^+^CD11b^-^, CD27^+^CD11b^+^, and CD27^-^CD11b^+^ subpopulations. In the development of NK cells, the double negative subset represents the naive cells that produce cytokines, while the single CD11b positive subset represents the mature cells with cytotoxic functions, and the CD27 positive subsets were considered intermediate subtypes (56, 57). We found a significant increase in peripheral NK cells in GDM model mice, particularly CD27^-^CD11b^+^ cells, and an increased proportion of CD56^dim^CD16^+^, CD226^+^, NKG2D^+^, CD27^-^CD11b^+^ NK cells in GDM patients, suggesting that cytotoxic NK cells are predominant in circulation. It is consistent with findings in previous human studies, which found an increase in CD56^dim^CD16^+^ NK cells and a decrease in CD56^bright^CD16^-^ subtype NK cells (14, 15, 50). Trembath et al. reported that NKG2D signaling in the pancreas is likely relevant to type 1 diabetes pathogenesis (58), and the function of active CD8^+^ T cells was increased by the NKG2D signal pathway. Therefore, we propose that the aberrant NK cell subtypes in GDM may also be related to the NKG2D signaling pathway, the interaction of NKG2D- NKG2D ligands may be a checkpoint to NK cell dysfunction in GDM patients. However, we found that NK cells located in the maternal-fetal interface were significantly reduced in GDM dams, particularly the CD27^-^CD11b^-^ subset. After pregnancy, NK cells significantly increased aggregation between maternal-fetal interfaces (17, 59), and was demonstrated that CD27^-^CD11b^-^ subtype NK cells were the most prevalent subpopulation of NK cells in the decidua, which secreting growth-promoting factors and contributing to fetal nutrition (24). Similarly, we found the proportion of decidual NK cells were significant related to fetal weight especially CD27^-^CD11b^-^ subsets. We hypothesize the main cause of fetal undernutrition in GDM mice may be the deficiency of CD27^-^CD11b^-^ NK cells in the decidua. Nonetheless, further research is required to determine the mechanism by which hyperglycemia affected the changes in the subtype of NK cells resulting in fetal growth restriction.

It is likely that the placental environment selectively differentiates the NK cell population in GDM, affecting the health of the fetus. In recent studies, it has been demonstrated that cytotoxic NK cells are more dependent on glucose metabolism than regulatory NK cells (36), suggesting that a high glucose environment may increase glycolysis and oxidative metabolism, thus causing NK cells to differentiate into cytotoxic subsets. In order to further clarify the differentiation pathway of NK cells at the maternal fetal interface, we reanalyzed the scRNA-Seq dataset obtained from Yang et al. (60) and focused primarily on NK cell differentiation (**Supplementary Figure 4**). GDM patients had significantly higher percentages of NK1 (enriched in natural killer cell-mediated cytotoxicity) in their placentas than controls, consistent with our findings in mice. The differentiation of NK1 to NK3 (enriched in ferroptosis) is reduced. Differentiation related genes were tested, and annexin A1, C-X-C motif chemokine ligand 8 (CXCL8), and CD52 expression was significantly reduced in NK cells of GDM patients, while the expression of C-C motif chemokine ligand 3 (CCL3) was significantly increased. Allen et al. (61) reported that CCL3 enhances antitumor immune priming with dependency on NK cells, Strunz B. et al. (62) provided evidence that less differentiated uterine NK cells were the proinflammatory subset capable of producing IFN-γ, CCL3, CCL4, and TNF. We believe that hyperglycemia inhibits the differentiation of decidual cytotoxic NK cells into other subsets, leading to excessive production of pro-inflammatory cytokines and adverse pregnancy outcomes. In spite of this, there is no clear consensus regarding whether CCL3 is the key point and how it effects NK cells differentiation. NK cell differentiation is one of the directions worth exploring in our future research, since it may help to better understand the changes in NK cells during pregnancy and further elucidate the reasons for adverse pregnancy outcomes caused by NK cell changes in GDM.

The safety and effectiveness of immunotherapy for diabetes have been reported, and the therapeutic effect of Treg cell infusion in type 1 diabetes patients has been confirmed in mice and humans (63–65). However, there were deficient reports on the application of immunotherapy for GDM patients. Our study demonstrates a significant decrease in the proportion of NK cells in peripheral blood, accompanied by an increase in decidual CD27^-^CD11b^-^ NK cells after intrauterine NK cell immunotherapy. This was associated with improved fetal weight on GD14.5, suggesting that intrauterine therapy may regulate high glucose-induced changes in NK cell function. Nowadays, intrauterine immune cell infusion has been attempted and applied to improve adverse pregnancy outcomes. For example, intrauterine infusion of Treg cells may increase the live birth rate of recurrent miscarriage patients (66), and intrauterine infusion of PBMC or granulocyte colony-stimulating factor may increase the embryo implantation rate of recurrent implantation failure patients (67, 68). In our study, intrauterine infusion of NK cells was shown to improve the growth restriction of the fetus in GDM, which may be associated with the involvement and regulation of immune remodeling by donor NK cells or cytokines at the maternal-fetal immune interface, reducing the cytotoxic effect of NK cells in high glucose environments, as well as protecting and nourishing the fetus. We have administered intrauterine NK infusions at two time points: GD2.5 and GD5.5. Unfortunately, both normal pregnancy mice and GDM mice lost their pregnancies when treated with NK cells at GD5.5 (not shown). To avoid the impact of immunotherapy on embryo implantation, immunotherapy or intrauterine infusion therapy is normally administered before pregnancy or in the very early stages of pregnancy in clinical practice (66, 69, 70). Therefore, performing intrauterine infusion at a very early stage is safer and will have a smaller impact on the miscarriage rate of embryos.

Our study provides a relatively comprehensive demonstration of the effects of GDM on NK cell profile, suggesting that maternal hyperglycemia promotes NK cell differentiation into cytotoxic subpopulations. Additionally, we partially improved the adverse effects of hyperglycemia on pregnancy through intrauterine NK cell infusion. However, there are still areas where our findings need further refinement. Firstly, the non-obese GDM mouse model is not suitable for all GDM patients, and it has a higher tendency to affect non-obese (BMI ≤ 24kg/m^2^) individuals, those who have fetal growth restriction or low birth weight, and those who have reduced fetal growth. Secondly, the specific mechanism by which changes in NK cell subtypes result in adverse pregnancy outcomes has not been clarified, which will be the focus of our future research. Thirdly, changes in nourishing cytokines and other immune cells have not been detected at the maternal-fetal immune interface after intrauterine NK cell infusion, and the specific mechanism of immunotherapy is unclear. Future experimental designs will be necessary to further explore this issue. Fourthly, despite each fetal weight, the average fetal weight of each dam was similar between two groups after intrauterine reinfusion of NK, this may have a bias due to the small sample number. Fifthly, as for human work, NK cell composition was only investigated in blood. Sixthly, the protocol for STZ-induced diabetes mostly induces a mice form of type 1 diabetes, while GDM is more likely to type 2 diabetes, characterized by elevated levels of insulin, which has certain limitations in explaining the mechanism of GDM. Moreover, we obtained purified NK cells from the donor dam’s spleen. Splenic NK contains complex NK cell subpopulations, and it would be better to treat with purified CD27^-^CD11b^-^ NK cells only. Lastly, in our study, no adverse reactions were observed in the mother mice after intrauterine immunotherapy, however, its clinical application still requires more rigorous evaluation of safety, efficacy, and dosage.

In summary, the present study identified the proportion and phenotype of NK cells in peripheral blood and the maternal-fetal interface of non-obese GDM mouse models, which may provide therapeutic options for improving adverse pregnancy outcomes of GDM patients.

## Data availability statement

The original contributions presented in the study are included in the article/**Supplementary Material**. Further inquiries can be directed to the corresponding authors.

## Ethics statement

The studies involving humans were approved by institutional review board at Tangdu Hospital affiliated with Air Force Medical University. The studies were conducted in accordance with the local legislation and institutional requirements. The participants provided their written informed consent to participate in this study. The animal study was approved by Ethics review Board of Air Force Medical University. The study was conducted in accordance with the local legislation and institutional requirements.

## Author contributions

YX: Conceptualization, Data curation, Formal analysis, Methodology, Project administration, Writing – original draft, Writing – review & editing. YW: Conceptualization, Data curation, Formal analysis, Methodology, Validation, Writing – review & editing. MW: Data curation, Investigation, Methodology, Software, Validation, Writing – review & editing. SC: Conceptualization, Project administration, Writing – review & editing. HL: Data curation, Methodology, Writing – review & editing. HM: Data curation, Resources, Writing – review & editing. HY: Data curation, Investigation, Software, Writing – review & editing. YH: Formal analysis, Methodology, Writing – review & editing. KT: Investigation, Methodology, Writing – review & editing. XC: Investigation, Visualization, Writing – review & editing. JD: Formal analysis, Validation, Writing – review & editing. XW: Funding acquisition, Supervision, Writing – review & editing. LC: Conceptualization, Funding acquisition, Investigation, Project administration, Supervision, Writing – review & editing.
