# Supplementary material for: Aberrant NK cell profile in gestational diabetes mellitus with fetal growth restriction
Source: Front Immunol. 2024 Feb 5;15:1346231. doi: 10.3389/fimmu.2024.1346231 (PMC10875967; doi:10.3389/fimmu.2024.1346231)
Supplement: Supplementary file 1 [file DataSheet_1.docx]

Supplementary Material

# Supplementary Figures and Tables

## Supplementary Figures


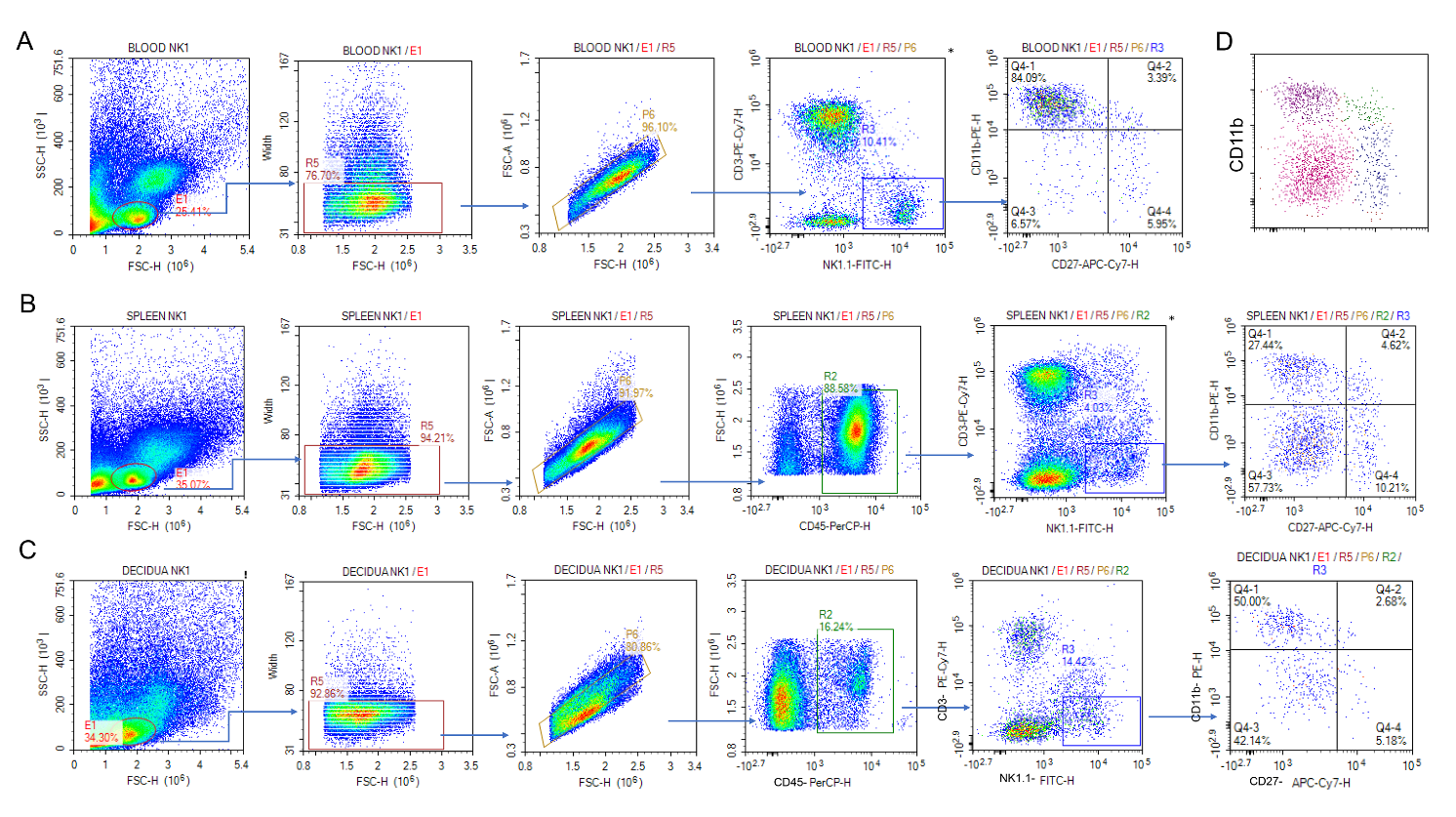


Supplementary Figure 1. Gating strategy for flow cytometry experiments of NK cells and their subpopulation in blood (A), spleen (B), and decidua (C). Lymphocytes were gated by SSC-H and FSC-H, as indicated, and then the adhesive cells were removed by FSC-H vs. width and vs. FSC-A. Tissue leukocytes were further gated with CD45 vs. FSC-H and NK cells were gated with CD3^-^NK1.1^+^. Finally, four NK cell subsets were gated by CD27 and CD11b. (D) Scheme of the NK cell subsets grouping.


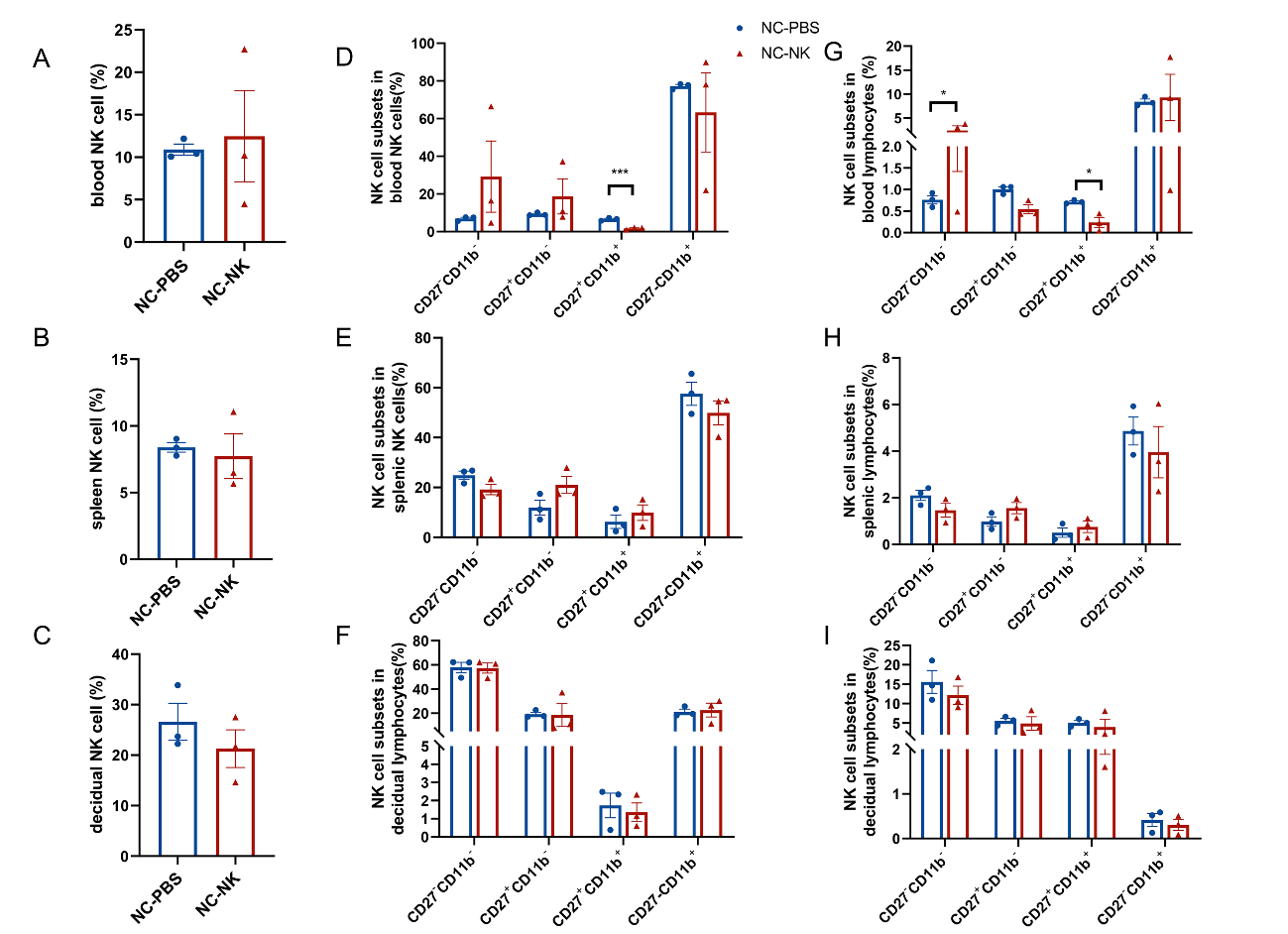


Supplementary Figure 2. Effect of NK cell immunotherapy on NK cells and NK cell subsets in normal pregnancy. (A-C) No difference was found in blood, spleen, and decidua NK cells with or without treatment in normal pregnancy. (D-F) Quantitative results for NK cell subsets in total NK cells in blood, spleen, and decidua. (G-I) Proportion of NK cell subsets in total lymphocytes in blood, spleen, and decidua. n=3 each group. Data are presented as mean ± SEM. *p < 0.05, ***p<0.001.


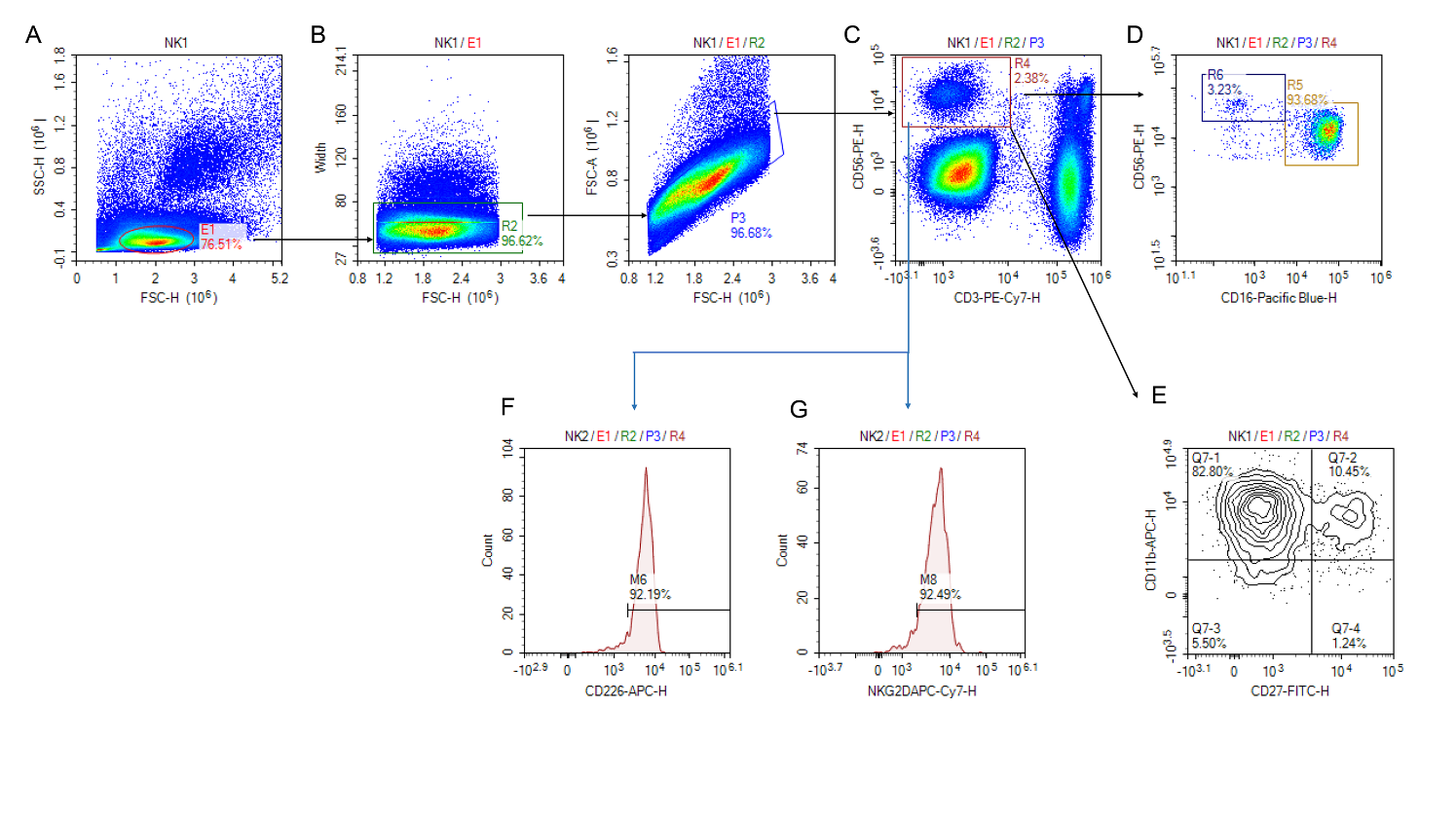


Supplementary Figure 3. Gating strategy for flow cytometry experiments of NK cells and their subpopulation in blood in humans. PBMC were gated by SSC-H and FSC-H, as indicated (A), and then the adhesive cells were removed by FSC-H vs. width and vs. FSC-A (B). NK cells were gated with CD3-CD56+(C). NK cell subsets were categorized with CD56 and CD16 (D), CD27 and CD11b (E). CD226 positive NK cells were gated in F and NKG2D positive NK cells were gated in G.


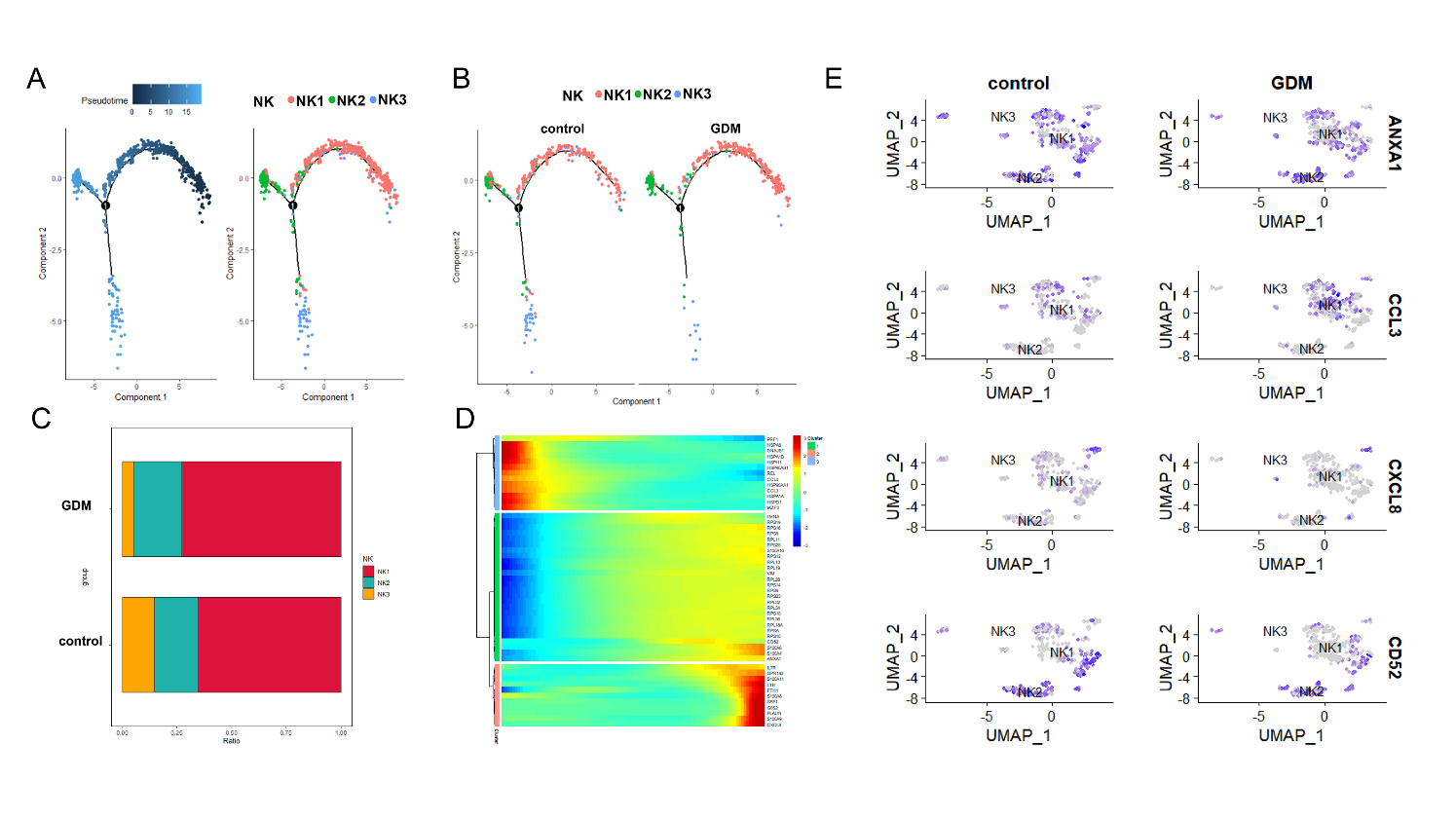


Supplementary Figure 4| Pseudo-Time analysis of clusters and selected marker genes. (A) Distribution of cells in each cluster on the pseudo-time trajectory. (B) Pseudo-time trajectory of single-cell transcriptomics data colored according to cluster from GDM and control NK cells. (C) Comparison of NK cell cluster composition between two groups of patients. (D) Heatmap of gene markers in NK cell differentiation process by Pseudo-time analysis. (E) Representative differential gene expressions in NK cell clusters in control and GDM patients.
